# The influence of robotic assistance on hidden blood loss in the treatment of posterior pelvic ring injuries with sacroiliac screw fixation: A retrospective cohort study

**DOI:** 10.1097/MD.0000000000051274

**Published:** 2026-10-30

**Authors:** Ding Xu, Jing Guo, Weigang Lou, Ming Li, Guanyi Liu, Yunqiang Zhuang, Jianming Chen

**Affiliations:** a Department of Orthopedic Trauma Surgery, Ningbo No. 6 Hospital, Ningbo, Zhejiang, China; b Department of Traumatic Orthopedics, Ningbo Clinical Research Center for Orthopedics, Sports Medicine & Rehabilitation, Ningbo, Zhejiang, China; c Department of Orthopedic Trauma Surgery, The Second Hospital of Jilin University, Changchun, China.

**Keywords:** HBL, posterior pelvic ring injury, Sacroiliac screw, surgical robots

## Abstract

Pelvic posterior-ring injury is a severe traumatic condition accompanied by substantial perioperative hidden blood loss (HBL). Minimally-invasive sacroiliac screw fixation is widely applied for this injury, yet postoperative anemia remains common and is predominantly caused by HBL. Whether robotic-assisted screw placement can reduce HBL in patients with posterior pelvic-ring injuries remains unclear. We retrospectively analyzed the data of 64 patients with posterior pelvic ring injury treated with sacroiliac screw fixation in our hospital from January 2020 to June 2023. Based on the use of intraoperative robot assistance during surgery, the 64 patients were divided into 2 groups. The total blood loss (TBL) and HBL were compared to analyze the difference between the 2 groups. In the robotic surgery group, TBL was 335.51 ± 31.46 mL, and the HBL was 300.96 ± 37.01 mL. In the freehand group, the TBL was 410.57 ± 54.55 mL, and the HBL was 370.34 ± 54.51 mL. The difference between the 2 groups was statistically significant (*P* < .001). However, there was no statistically significant difference in visible blood loss (VBL) and surgical duration between the 2 groups (*P* > .05). The robotic group presented a significantly smaller postoperative maximum hemoglobin drop (*P* < .001), whereas transfusion-related indicators were comparable between the 2 groups (*P* > .05). In the treatment of posterior pelvic ring injuries with sacroiliac screws, the assistance of surgical robots plays a significant role in reducing intraoperative blood loss, particularly hidden blood loss. Compared to traditional freehand screw placement, we recommend considering the incorporation of robotic assistance during surgery.

## 1. Introduction

Since percutaneous sacroiliac screw fixation (PSSF) was introduced for therapeutic interventions for lesions affecting the posterior pelvic ring in 1989, it has become the “gold standard” for managing these types of injuries.^[1]^ PSSF offers numerous benefits, including a reduced risk of infection, less intraoperative blood loss, and is recognized as a minimally invasive and effective treatment option.^[2]^ Some researchers even argue that PSSF is the only minimally invasive technique capable of restoring stability to lesions affecting the posterior pelvic ring.^[3]^ Compared to traditional open surgical reduction followed by internal fixation, PSSF is less invasive, involves shorter surgical times, and allows for quicker recovery.^[4]^ Even surgical placement of sacroiliac screws via percutaneous approach for lesions affecting the posterior pelvic ring, patients may still encounter varying levels of postoperative anemia, which can hinder recovery, increase healthcare costs, and raise mortality rates.^[5]^ Xu et al reported that nearly 100% of patients treated with minimally invasive sacroiliac screw fixation developed postoperative anemia.^[6]^ This finding challenges the general assumption that minimally invasive techniques result in less bleeding and faster recovery. While clinicians tend to focus on visible blood loss, the significance of hidden blood loss (HBL) is often underestimated. Studies have shown that HBL accounts for over 90% of the total blood loss (TBL) following minimally invasive fixation using sacroiliac screws for lesions affecting the posterior pelvic ring, making it the primary cause of postoperative anemia in these patients.^[6]^ This issue significantly affects the prognosis of pelvic trauma patients, particularly the elderly or those with multiple comorbidities. With the growing use of surgical robots in clinical practice, the technique of PSSF has undergone significant improvements, reduced surgical complexity, and enhanced precision and safety.^[7-9]^ However, the impact of robotic assistance on blood loss, particularly HBL, in treating posterior pelvic ring injuries with PSSF has not been reported. In this study, we retrospectively analyzed cases from January 2020 to June 2023 at our hospital, where robotic assistance was used for Sacroiliac screw stabilization in managing pelvic injuries. We examined perioperative bleeding, HBL, and discussed how robotic assistance influences perioperative HBL to offer valuable insights for clinical therapeutic and preventive approaches.

## 2. Participants and procedures

### 2.1. Patients

This study conducted a retrospective analysis of the medical records of Individuals treated with PSSF for Lesions affecting the posterior pelvic ring at our institution between the start of January 2020 and the end of June 2023. The criteria for inclusion in the study: patients clinically diagnosed with fresh Lesions affecting the posterior pelvic ring and underwent PSSF (injury to surgery time ≤ 3 weeks); pure sacroiliac screw fixation without additional procedures such as anterior fixation (excluding staged external fixator procedures); fractures categorized under AO/OTA types 61B, 61C1.2, and 61C1.3^[10]^; preoperative and postoperative complete blood count examination; Patients with no prior thrombosis, coagulation abnormalities, or other bleeding conditions associated with elevated risk; no history of blood transfusions before surgery or long-term use of anticoagulant drugs; fluid replacement within 24 hours after surgery not exceeding 2000 mL^[11]^; complete clinical data (such as visible blood loss, Hct records, surgical duration, time to surgery, etc). Initially, 122 cases were considered for the study; however, based on the inclusion criteria, 58 patients were excluded. Among them, 50 patients underwent pelvic anterior ring plating simultaneously with sacroiliac screw fixation, 3 patients received more than 2000 mL of fluid replacement within 24 hours post-surgery, 3 had incomplete clinical records, and 2 underwent preoperative blood transfusions. Sixty-four patients were ultimately enrolled in the study, aged between 27 and 62 years, with a mean age of 43.65 ± 4.45 years. The patients were grouped into 2 categories according to robotic assistance was used during surgery. The robotic surgery group consisted of 29 patients (17 males, 12 females), aged 27 to 59 years, with a mean age of (42.85 ± 4.10) years. The freehand surgery group included 35 patients (20 males, 15 females), aged 29 to 62 years, with an average age of (44.34 ± 5.08) years. All patients provided routine preoperative written informed consent for surgical treatment. Individual informed consent for this retrospective data analysis was waived by the institutional ethics committee owing to the anonymized non-interventional nature of this study.

### 2.2. Surgical procedure

The surgery was typically carried out under either general anesthesia or a continuous epidural block, Based on the individual’s medical condition and the requirements of the surgery. The choice of anesthesia was made to provide optimal comfort and safety throughout the operation. Once anesthesia was administered, the patient was laid supine on a carbon fiber surgical platform, and the surgical area was sterilized and draped as part of the preoperative preparation. For the freehand surgery group, a 1 cm long, longitudinal incision was performed approximately 4 cm away from the upper posterior iliac crest, and the underlying tissue was dissected to expose the ileum, where a positioning sleeve was placed. A 2 mm diameter guide pin was chosen and inserted perpendicular to the back portion of the iliac wing. Fluoroscopic guidance with a Mobile x-ray unit (Pelvic inlet, outlet, and side-view x-rays) was used throughout the process. The guide pin navigated through the sacroiliac joint, crossed the sacral auricular surface, and penetrated the sacral wing, before reaching the S1 vertebra between the sacral hiatus and the anterior opening of the sacrum. During insertion, the guide pin was kept parallel to the sacral promontory and inclined approximately 25 degrees from posterior to anterior relative to the frontal plane of the pelvis separating the body into front and back sections. Typically, the guide pin was inserted to a depth of around 8 cm, stopping near the midline of the sacral vertebral body. A hollow tension screw (Wego Medical Instruments Co., Ltd.) was then inserted along the guide pin. For the robotic surgery group, the Tirobot Orthopedic Robotic Surgery System (TINAVI Medical Technology Co., Ltd.) was employed. After confirming the proper functioning of the robotic equipment, sterile draping was applied. Following the routine disinfection and draping of the surgical field, A locator marker was affixed to the iliac wing of the unaffected side, and the positioning scale was fine-tuned. The mobile X-ray machine was used to capture images of the pelvis, obtaining images from the outlet, inlet, and lateral views, which were transmitted to the robotic console for target screw planning. The robotic arm was then used to guide the entry and placement direction of the guide pin according to the planned path. The practitioner created a 1 cm surgical opening based on the robotic arm’s guidance, cut the fascia, and inserted the guide pin sleeve into the bone surface after separating the muscle tissue. A hollow tension screw of appropriate length (Wego Medical Instruments Co, Ltd) was inserted along the sleeve once the guide pin placement was confirmed.

### 2.3. Postoperative care

Antibiotics should be administered within 24 hours after surgery to prevent infection. X-rays and blood tests should be performed, and blood transfusions and other relevant treatments should be administered based on the patient’s anemia status. Non-weight-bearing activities for the lower limb may commence on the second day following surgery. Four weeks postoperatively, the patient can start ambulating out of bed, progressively increasing weight-bearing on the affected limb during walking.

### 2.4. Calculation of blood volume indices

To minimize errors, 1 person conducted the measurement 3 times for each relevant indicator, and the average value was used. Hemoglobin (HB) levels were evaluated to identify the presence of anemia. Anemia, as defined by the World Health Organization, occurs when hemoglobin levels drop below 130 g/L in males and 120 g/L in females.^[12]^ To determine the patient’s blood volume (BV), the formula proposed by Nadler et al^[13]^ was used.

BV is calculated by multiplying the height (*H*) raised to the third power by *k*1, adding the body weight (*W*) multiplied by *k*2, and then adding *k*3.

In the formula used to calculate blood volume (BV), several variables are taken into account. BV represents the preoperative blood volume, which is measured in liters. The equation also factors in the patient’s height, denoted as *H*, which is recorded in meters, and body weight, represented by *W*, measured in kilograms. These key variables are adjusted by specific constants that vary based on the patient’s sex. For male individuals, the constants are set as *k*1 = 0.3669, *k*2 = 0.03219, and *k*3 = 0.6041, ensuring a more accurate estimation of blood volume. On the other hand, for female individuals, the constants are slightly different, with *k*1 = 0.3561, *k*2 = 0.03308, and *k*3 = 0.1833. This differentiation accounts for physiological differences between males and females, allowing the formula to provide a tailored estimation of blood volume for each individual based on both their physical characteristics and sex. The use of these constants is critical for obtaining accurate calculations, which are important for assessing patients’ blood volume in clinical settings.

Total blood loss (TBL) is an important metric in evaluating blood loss during surgery, particularly for procedures with significant hemorrhage risk. TBL is calculated by assessing the volume of red blood cells lost, determined by the difference in hematocrit (Hct) levels before and after surgery. Hematocrit represents the percentage of blood volume made up of red blood cells. The formula for calculating the volume of red blood cells lost is: Volume of red blood cells lost = BV × (Preoperative Hct − Postoperative Hct) + amount of blood transfused. Here, BV refers to the patient’s blood volume, which is estimated based on height, weight, and sex, while the difference in Hct values reflects the loss of red blood cells.

Once the volume of red blood cells lost is calculated, TBL is obtained by dividing this loss by the average hematocrit (Hct mean).^[14]^ The Hct mean is calculated as: Hct mean = 1/2 × (Preoperative Hct + Postoperative Hct), where preoperative Hct is typically measured on the second morning after admission, and postoperative Hct is taken on the third morning after surgery. This average helps adjust for changes in hematocrit due to factors such as fluid infusion or transfusions.

Visible blood loss (VBL) refers to the blood lost during surgery that is observable. VBL is calculated by adding fluid collected in the suction device and the blood absorbed by surgical gauze, subtracting the volume of irrigation fluid used. Hidden blood loss (HBL), which is not immediately apparent, is calculated by subtracting VBL from the TBL. HBL is crucial for understanding blood loss that occurs internally, which can affect postoperative recovery and management.

We also recorded 3 additional perioperative transfusion-related outcomes: postoperative maximum hemoglobin drop (MHD) was defined as the largest difference between preoperative hemoglobin value and any postoperative hemoglobin measurement within 7 days after surgery. Postoperative transfusion rate referred to the proportion of patients receiving allogeneic blood transfusion within the postoperative hospitalization period. Total RBC transfusion volume represented the cumulative volume of allogeneic red blood cell products administered during postoperative hospitalization.

### 2.5. Analysis of statistics

Sample-size calculation was performed using published HBL mean and standard deviation from posterior pelvic-ring injury patients; α = 0.05, β = 0.2 (power = 80 %). A 10% loss-to-follow-up was considered. Multivariate linear regression was used to adjust for confounding factors including surgeon experience, pelvic injury severity, number of implanted sacroiliac screws, and intraoperative hemostatic interventions. SPSS 20.0 was employed for the statistical analysis. For continuous variables that follow a normal distribution and have homogeneity of variance, an independent sample Student *t* test can be used for comparison; When the variance is uneven, Welch’s *t* test is used. Continuous variables that do not follow a normal distribution are subjected to Mann–Whitney *U* test. The categorical variables were tested using Fisher exact test. A *P*-value below .05 was considered indicative of a significant difference. All statistical methods were selected to ensure accurate and reliable comparisons between the 2 groups, facilitating a comprehensive evaluation of the study results.

## 3. Results

No significant statistical differences were observed in baseline characteristics, including gender, age, injury cause, classification, and time to surgery, between the 2 groups (*P* > .05; Table 1). All 64 patients completed follow-up, with the robotic surgery group having a follow-up period ranging from 10 to 22 months (mean: 13.0 ± 1.2 months), and the freehand surgery group having a follow-up duration from 9 to 20 months (mean: 12.0 ± 1.3 months). No complications such as infection, screw loosening, and internal fixation failure were found in either group of patients during postoperative follow-up.

**Table 1:** Comparison of basic characteristics of cases.

	Gender (m/f)	Age (yr)	BMI (kg/m^2^)	Cause of injury (case)		Classification (AO/OTA) (case)			Time to surgery (d)
				Traffic accident	Fall injury	61b	61c1.2	61c1.3	
Surgical robot group	17/12	42.85 ± 4.10	23.28 ± 2.14	10	19	20	7	2	3.1 ± 1.8
Freehand group	20/15	44.34 ± 5.08	23.80 ± 2.36	14	21	23	9	3	3.3 ± 1.5
*T* value	–	0.345	0.447	–		–			0.333
*P* value	.551	.747	.564	.473		.395			.716

BMI = body mass index.

In the robotic surgery group, total blood loss (TBL) averaged 335.51 ± 31.46 ml, and hidden blood loss (HBL) was 300.96 ± 37.01 mL. In the freehand group, TBL was 410.57 ± 54.55 mL, and HBL was 370.34 ± 54.51 mL. A significant statistical difference was detected between the 2 groups (*P* < .001). However, no significant differences were noted in visible blood loss (VBL) or surgical duration across the groups (*P* > .05; Table 2).

**Table 2:** Comparison of blood loss-related indicators between the 2 groups.

	Calculated total blood loss (mL)	Visible blood loss (mL)	Hidden blood loss (mL)	Surgical duration (min)	Max HB drop (g/L)	PTR (%)	TTV (mL)
Surgical robot group	335.51 ± 31.46	37.48 ± 6.09	300.96 ± 37.01	65.65 ± 8.80	20.3 ± 6.1	2 (6)	13.79 ± 51.75
Freehand group	410.57 ± 54.55	40.22 ± 5.29	370.34 ± 54.51	62.88 ± 5.80	27.9 ± 6.3	3 (8)	20.00 ± 60.73
*T* value	−6.875	−1.928	−6.035	1.452	−4.94	/	−0.442
*P* value	<.001	.058	<.001	.153	<.001	1.000	**.660**

HB = hemoglobin, PTR = operative transfusion rate, TTV = total RBC transfusion volume.

The data of TTV presents a skewed distribution with some extreme values. We used mean ± standard deviation for consistent presentation with other indicators in this table, and nonparametric test was applied for intergroup comparison. The bold value is the *P* value for TTV comparison (*P* = .660 > .05), showing no statistically significant difference.

Postoperative MHD, postoperative transfusion rate, and total RBC transfusion volume were further compared between groups. After multivariate adjustment for predefined confounders (surgeon experience, severity of pelvic injury, number of implanted sacroiliac screws, and intraoperative hemostatic interventions), the comparative results are summarized in Table 2. The robotic‑assisted group exhibited a significantly smaller maximal postoperative Hb drop than the freehand group (*P*<.001). No significant inter‑group differences were observed in postoperative transfusion rate and total RBC transfusion volume (*P*>.05).

## 4. Discussion

HBL has long been an easily overlooked clinical phenomenon. The earliest literature report was by Pattison et al in 1973, who observed it after knee replacement surgery.^[15]^ It was formally defined as HBL in 2000.^[16]^ Studies have shown that the overall blood loss following total knee arthroplasty ranges from 500 to 1500 mL, with the amount of HBL accounting for about half of this.^[17,18]^ In spinal fusion surgery, the literature reported that HBL comprised approximately 42% to 47% of the total blood loss.^[19,20]^ Similarly, HBL has also been reported in hip surgeries, with the percentage fluctuating between 50% and 80%.^[21,22]^ There was limited research on HBL related to pelvic injuries, particularly with traditional percutaneous sacroiliac screw fixation, which was often considered a minimally invasive procedure.^[23]^ Nonetheless, research by Xu et al revealed that over 50% of patients with posterior pelvic ring injuries exhibited varying degrees of anemia before surgery, and the incidence of anemia after minimally invasive sacroiliac screw treatment was close to 100%.^[6]^ In fact, the amount of HBL can exceed 90% of the total blood loss. During sacroiliac screw treatment of posterior pelvic ring injuries, there was very little apparent blood loss throughout the surgery, which often led doctors to overlook postoperative anemia in patients. Therefore, attention and emphasis on HBL in such surgeries urgently needed to be increased. With the clinical promotion and use of surgical robots, many studies have reported that robot-assisted surgery has numerous advantages over traditional freehand sacroiliac screw fixation, such as less trauma, reduced bleeding, less fluoroscopy, and higher accuracy.^[24-26]^ However, there were few reports on whether the intervention of surgical robots affects the amount of HBL during surgery.

This study found that the assistance of surgical robots significantly reduced intraoperative TBL and HBL through a retrospective analysis of cases where surgical robots assisted with sacroiliac screw treatment for posterior pelvic ring injuries. Statistical analysis revealed a significant difference across the 2 groups, highlighting distinct outcome variations when compared to the freehand group. In actual clinical practice, the freehand group’s one-time success rate for inserting guide pins was often lower, requiring repeated needle entry point and guide pin channel adjustments during surgery.^[27]^ This process easily created multiple screw channels within the bone marrow cavity, which became potential bleeding spaces and increased HBL. In contrast, surgery assisted by robots generally achieved one-time success in screw placement, with minimal need for repeated adjustments of the needle direction and depth.^[28]^ Additionally, the duration from skin incision to the completion of screw placement was notably shorter in the robotic-assisted group compared to the freehand group, which was another crucial factor in the significant reduction of TBL and HBL.^[29]^ No notable differences were observed between the 2 groups in terms of VBL and surgical duration. Percutaneous screw placement was inherently a minimally invasive procedure with small incisions and minimal visible blood loss. Therefore, the VBL difference between the 2 groups was minor. In the robot-assisted group, preoperative procedures included C-arm imaging and processing of qualified imaging data to plan the needle path, which were time-consuming. However, the subsequent screw placement was relatively straightforward, following the preplanned track safely and efficiently, with a success rate close to 100% for a single screw insertion. Once the planning was complete, the entire screw placement process required almost no fluoroscopy, only a single verification fluoroscopy after screw placement. In the freehand group, although there were no preoperative planning steps, the success rate of single screw insertion was lower, often requiring repeated adjustments and extensive fluoroscopy, thus spending more time during the screw placement phase. The study results also indicated that the assistance of surgical robots did not significantly speed up the surgery or reduce the operation time. However, it should be noted that as familiarity with the preoperative planning operation of the robot increases, the time it occupies will decrease, potentially leading to shorter surgery times. Although the robotic-assisted group achieved a lower postoperative MHD, there was no significant difference in postoperative transfusion rate and total transfusion volume between the 2 groups. This may be attributed to the overall low transfusion threshold compliance and small absolute transfusion volume in this cohort of relatively young trauma patients.

Our study was a retrospective analysis conducted at a single center, focusing on a relatively small sample of patients. Owing to the restricted sample size, the findings may not fully represent broader populations, and the results should be interpreted with caution, considering the study’s design and scope. It lacked the benefits of multi-center collaboration, larger datasets, and high-quality randomized controlled trials (RCTs) that could provide more comprehensive clinical guidance. As a retrospective study, our analysis was subject to selection bias. We performed multivariate regression to adjust for surgeon experience, pelvic injury severity, number of implanted screws, and intraoperative hemostatic interventions to mitigate confounding bias. Specifically, the experience level of the surgeons was not uniformly recorded or adjusted for, which may have influenced the surgical outcomes. For example, more experienced surgeons may have preferentially performed robotic procedures, potentially overestimating their benefits. Future studies should incorporate surgeon experience as a confounding variable and consider prospective or randomized designs for validation. Differences in the number of intraoperative fluoroscopies can also be included in future research, which is of significant importance to both patients and surgeons.

Furthermore, posterior pelvic ring injuries are regularly seen with anterior ring injuries and surgical interventions, which complicates the collection of cases that meet the specific criteria for isolated posterior ring injuries. Therefore, we included cases where anterior ring external fixator surgery was performed in stages in our study. Although external fixation was minimally invasive and typically associated with negligible intraoperative bleeding, it may contribute to minor hidden blood loss due to soft tissue and periosteal injury. To evaluate its impact, subgroup and multivariate analyses were performed. The results indicated that external fixation did not significantly affect HBL (*P* > .05), and thus its influence was considered negligible in the final model. Nevertheless, this was acknowledged as a potential limitation.

## 5. Conclusions

Robotic support in managing posterior pelvic ring injuries with sacroiliac screws is crucial for reducing intraoperative blood loss, particularly in terms of hidden blood loss. Compared to traditional freehand screw placement, we strongly recommend incorporating robotic assistance during surgery.

## Acknowledgments

We thank all clinical staff involved in collecting clinical data for this study. All individuals named in acknowledgments have granted permission to be acknowledged.

## Author contributions

**Data curation:** Jing Guo, Weigang Lou, Ming Li, Guanyi Liu.

**Formal analysis:** Weigang Lou, Guanyi Liu.

**Methodology:** Jing Guo, Ming Li, Guanyi Liu.

**Supervision:** Ding Xu, Jianming Chen.

**Validation:** Yunqiang Zhuang, Jianming Chen.

**Visualization:** Ding Xu, Yunqiang Zhuang, Jianming Chen.

**Writing – review & editing:** Ding Xu.
